# Interaction effect of courtyard building form and orientation on energy performance of hospitals in warm humid climate

**DOI:** 10.1038/s41598-026-40632-1

**Published:** 2026-02-19

**Authors:** Harshalatha, Shantharam Patil

**Affiliations:** Manipal School of Architecture and Planning, Manipal Academy of Higher Education, Manipal, Karnataka 576104 India

**Keywords:** Architectural design, Building form, Climate change, Energy performance index (EPI), Interactive effect, Orientation, Warm humid, Climate sciences, Energy science and technology, Engineering, Environmental sciences, Environmental social sciences

## Abstract

The built environment plays a crucial role in optimizing energy consumption within hospital buildings. Enhancing its efficiency is vital for sustainable development. The form, shape and orientation of hospital buildings significantly impact their energy performance, leading to energy savings, improved indoor air quality, and enhanced thermal comfort. This research focuses on assessing the interactive effect of courtyard building forms and orientation on energy performance in hospital buildings in warm humid climatic zone of Indian coastal region. The findings suggest that building form consistently plays a dominant role in determining the energy performance Index (EPI), while orientation exerts a more nuanced and season-dependent influence. The study reveals a significant interaction between forms and orientation, indicating an interdependent effect on energy-efficient building design. The model explains 98.6% of variation in EPI, with an excellent fit and low standard error, highlighting the importance of optimizing both forms and orientations for energy-efficient building designs. This study presents a novel perspective on performance-driven sustainable hospital design, tailored to the unique climatic demands of India’s coastal, warm-humid regions. In the context of climate change, the study emphasizes the need for passive design measures, adaptive architectural solutions, and performance-based modelling to lower operational energy demand and enhance resilience to climatic shifts.

## Introduction

One of the most significant challenges facing the modern world is getting buildings to utilize less energy in order to achieve sustainable development which meets the meet current needs while ensuring that environmental, economic, and social well-being are balanced across the entire life cycle of a project^1^. Buildings consume a significant portion of global energy, with 80% consumed during the operational stage and 20% during the construction stage, accounting for a significant portion of energy consumption worldwide in their daily operations and lifecycle^2^. The energy sector’s rapid growth due to population growth, industrialization, and urbanization has led to high carbon and greenhouse gas emissions. Per capita electricity consumption has increased, with energy demand rising 3.4% globally, with India being a major contributor. Buildings consume 48% of electricity, affecting energy security and access^3^.To improve efficiency, energy regulations must be developed and implemented at international, national, regional, and local level^4^. Passive design strategies are crucial for creating high-performance buildings, affecting energy efficiency and indoor environmental quality. The architectural form significantly impacts energy consumption, lighting, and various environmental factors including sun exposure, humidity, rain, wind, and shading^5^. The high energy consumption of buildings arises primarily from design and operational factors^6^. Building orientation significantly impacts thermal performance by reducing direct solar radiation exposure. It’s crucial to consider sun movement, latitude, and expected shading effect when selecting building direction^7^. Architects are encouraged to focus on building form and geometric configurations when designing medium height air-conditioned buildings, as this approach can lead to optimum shape and space placement, ultimately reducing cooling loads. Designing and constructing hospital buildings poses a complex challenge for specialists, particularly with energy performance as a primary goal^8^. The rising environmental and financial costs of energy have increased the focus on improving the energy performance (EP) of hospitals, which are crucial for commerce. Energy usage analysis in these facilities can enhance management practices. Architectural design plays a key role in energy performance, particularly in warm-humid climates like India, where building form affects airflow, sunlight exposure, and thermal comfort, thus reducing cooling and ventilation demands. Therefore, implementing passive design strategies such as natural ventilation, shading devices, and efficient spatial layouts is vital for sustainable hospital architecture in these regions^9,10^. In the post-monsoon season, effective ventilation and optimized building designs are crucial for managing high humidity, improving energy efficiency, and enhancing comfort in hospital buildings, which promotes sustainability and cost-effectiveness while positively impacting occupant health outcomes.

The built environment significantly influences the optimization of building energy consumption^11^. Improving the efficiency of the Built Environment is essential for sustainable development^12^. Building form significantly influences energy consumption by affecting the building’s interaction with its environment, affecting exposure to sunlight, humidity, rain, winds, shadows and self-shading. Studies have examined the impact of spatial form on energy consumption under various climatic conditions^13^. Hemsath and Alagheband examined several kinds of nonrectangular geometries, such as the L and U shapes, and assessed their advantages in terms of energy utilization and self-shading, which lowered summer solar heat gain^14^. Yeretzian et al., intended to determine the best building form for mitigating insolation and the effects of solar radiation on structures in an area where the majority of cooling methods involve applying passive design techniques to cool interior spaces^15^. Mohsenzadeh et al., investigated the impact of building form and self-shading on thermal performance, focusing on four basic geometric forms and determining the optimal form by controlling excessive solar radiation through self-shading for a reference building with fixed orientation^16^. Mirrahimi et al., found that optimal orientation increases daylight penetration while reducing artificial lighting needs. South and north orientations generally provide balanced daylight without excessive glare^17^. Nedhal et al., suggested that in hot-humid climates, east-facing orientations can result in glare and overheating, making proper shading critical^18^. ​Pathirana et al., examined the impact of building forms, zone sizes, and location on thermal comfort and energy consumption in naturally ventilated houses. Research shows that building form generally has a limited impact on thermal comfort and lighting energy, except for rectangular shapes with central staircases, which improve thermal comfort. Factors like zone sizes, locations, and window-to-wall ratio (WWR) are crucial, with WWR influencing thermal comfort by 20–55% ^19^. Building orientation has minimal effect. Future research will investigate both individual and collective factors related to these findings. By modelling energy and lighting usage through simulation strategy, Taleghani et al., also looked at the effects of residential structures on these metrics in the Netherlands. Therefore, by contrasting the three forms of one, two, and three-story single, linear, and central courtyard buildings, they discovered that the central courtyard form—which is thought to be the best mode for designing—was 22% less energy-consuming and had 9% less discomfort than the single form building. According to studies, utilizing a central courtyard may significantly save energy usage^19^. Ratti et al., did an analysis for the central courtyard form in the Moroccan hot climate and pointed out that it could be an optimal form to reduce energy consumption among building forms^20^. Tuhus-Dubrow and Krarti, developed a tool, used a genetic algorithm and building energy simulation engine to optimize building designs with minimal energy use. It examines various shapes, including trapezoids, L, T, cross, U, and H, and finds that rectangular and trapezoidal shapes have the lowest life-cycle costs^21^. Kurniawan et al., examines the impact of building form and window-to-wall ratio on thermal comfort and lighting energy consumption in tropical residential houses, highlighting the importance of EP assessment for architects and engineers^22^. Haseeb Q.S et al., simulated the energy consumption of six different building forms in Kirkuk, Iraq, over a year, finding that the T-shape model had the lowest energy consumption at a 285° rotation angle^23^. Almufarrej A.M et al., conducted the research in Kuwait to find the rectangular buildings with plan aspect ratios between 1:1.25 and 1:1.5 consume less energy when longer spans are oriented towards the north^24^. Shi Y et al., in their study in China revealed that outpatient buildings with grid courtyard forms have the highest energy-saving rate (16.3%) in cold climate zones^25^. A study on energy consumption in a general hospital by Shen et al., found that air-conditioning accounts for 40% of the total year’s energy consumption in a hot summer and cold winter zone^26^. Pacheco et al., found that building orientation, form, and the ratio between external surfaces and volume are the most significant factors influencing final energy demand. Optimized building forms and orientations significantly impact energy performance (EP) and can lead to energy savings^27^. However, understanding the relationship between building form, orientation, and energy consumption remains challenging, particularly for hospital buildings with courtyard forms. Previous studies have often modified other parameters alongside spatial form variables, complicating the assessment of the isolated effects of spatial form on energy consumption^13^. Recent literature shows combining of parametric building performance simulation with validated future weather dataset using tools such as EnergyPlus, Ladybug, and Latin Hypercube Samplin to evaluate envelope parameters across multiple climate scenarios. Global sensitivity methods (SRC and Sobol indices) consistently demonstrate that although absolute performance varies by downscaling method, supporting reliable early-stage design decisions under climate uncertainty^28^. A thorough framework for examining early-stage façade design choices under climate uncertainty is provided by this methodological integration, which combines validated climate data, high-resolution simulations, and rigorous sensitivity analysis^29^. For efficient parameter interaction analysis, recent developments in architectural parameter analysis currently include advanced statistical approaches including variance decomposition and distribution-based methodologies. The modern building performance analysis utilizes advanced uncertainty and sensitivity methods like Monte Carlo, ANOVA, and information-theoretic techniques to enhance energy prediction reliability and account for non-linear interactions. Research highlights the importance of seasonal energy performance dynamics, particularly in warm-humid regions, advocating for architects to consider seasonal variations to boost energy efficiency and occupant comfort, thus promoting climate-responsive design practices^30^. Empirical assessments examining the combined effects of building form and orientation in courtyard hospital buildings are significantly lacking. While substantial research has individually addressed either building orientation or form’s impact on energy performance (EP), studies analysing their interactive effects are scarce. Existing literature tends to isolate these parameters, focusing on static geometries or existing varied forms without considering their co-influence on energy outcomes. This gap is particularly pronounced in hospital buildings within India’s warm-humid climatic zones, where the relationship between form and orientation is crucial yet remains insufficiently explored. This research aims to address this deficiency by investigating how the synergy between building form and orientation affects EP in hospitals located in the coastal warm-humid regions of Karnataka state of India. Climates in warm humid regions show notable seasonal variations, influencing internal thermal loads and energy use. Analyzing hospital building form and orientation in relation to these changes can enhance energy efficiency. Compact building forms can lower cooling loads in summer, while proper orientation can improve natural ventilation during the monsoon and control solar heat gain in the post-monsoon period^31^. This paper explores how architectural design, particularly courtyard building form in relation to orientation and different seasons, can improve energy efficiency in hospitals located in warm-humid climates of India, highlighting the potential for sustainable, low-energy designs that enhance both environmental and operational outcomes through building energy simulation method. This research addresses a gap in existing literature by examining the combined and seasonal effects of courtyard building form and orientation on the energy performance and thermal comfort of hospitals in India’s coastal climate, an area that has not been extensively studied in sustainable design. The study aims to establish a climate-responsive form-generation framework that improves energy efficiency and occupant comfort in hospital buildings. It methodically investigates how varying courtyard configurations impact the Energy Performance Index (EPI) in specific coastal climatic conditions and assesses seasonal orientation variations alongside the interactive effects of form and orientation over time. The research integrates both temporal (seasonal) and spatial (form and orientation) climatic dynamics to go beyond conventional static design guidelines, proposing a performance-driven, context-specific approach to hospital architecture. The findings aim to inform an evidence-based design framework that advocates for sustainable, low-energy hospital development in India’s coastal regions, thereby providing valuable contributions to both practice and theory in climate-responsive healthcare design.

## Methodology

**Fig. 1:**
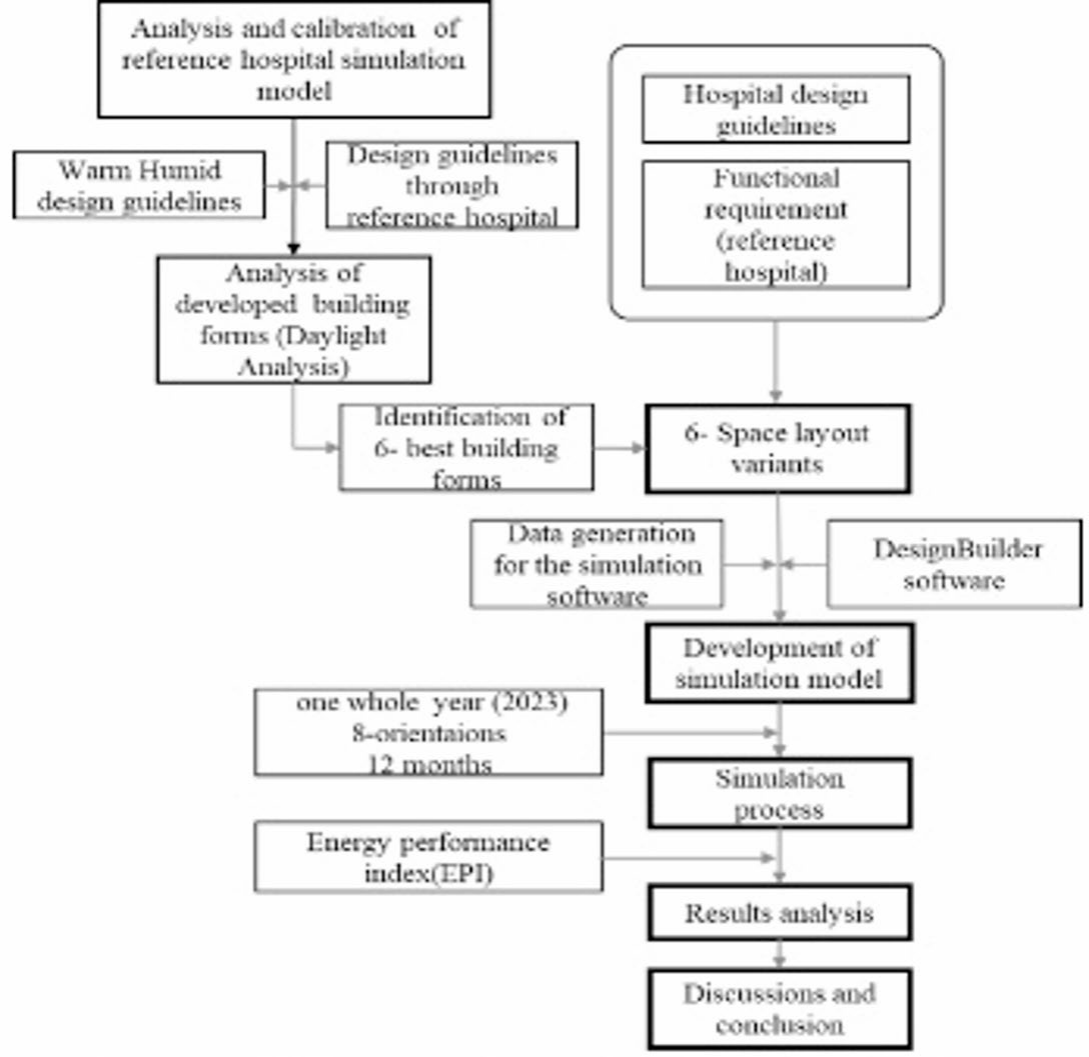
Detailed research methodology.

**Fig. 2:**
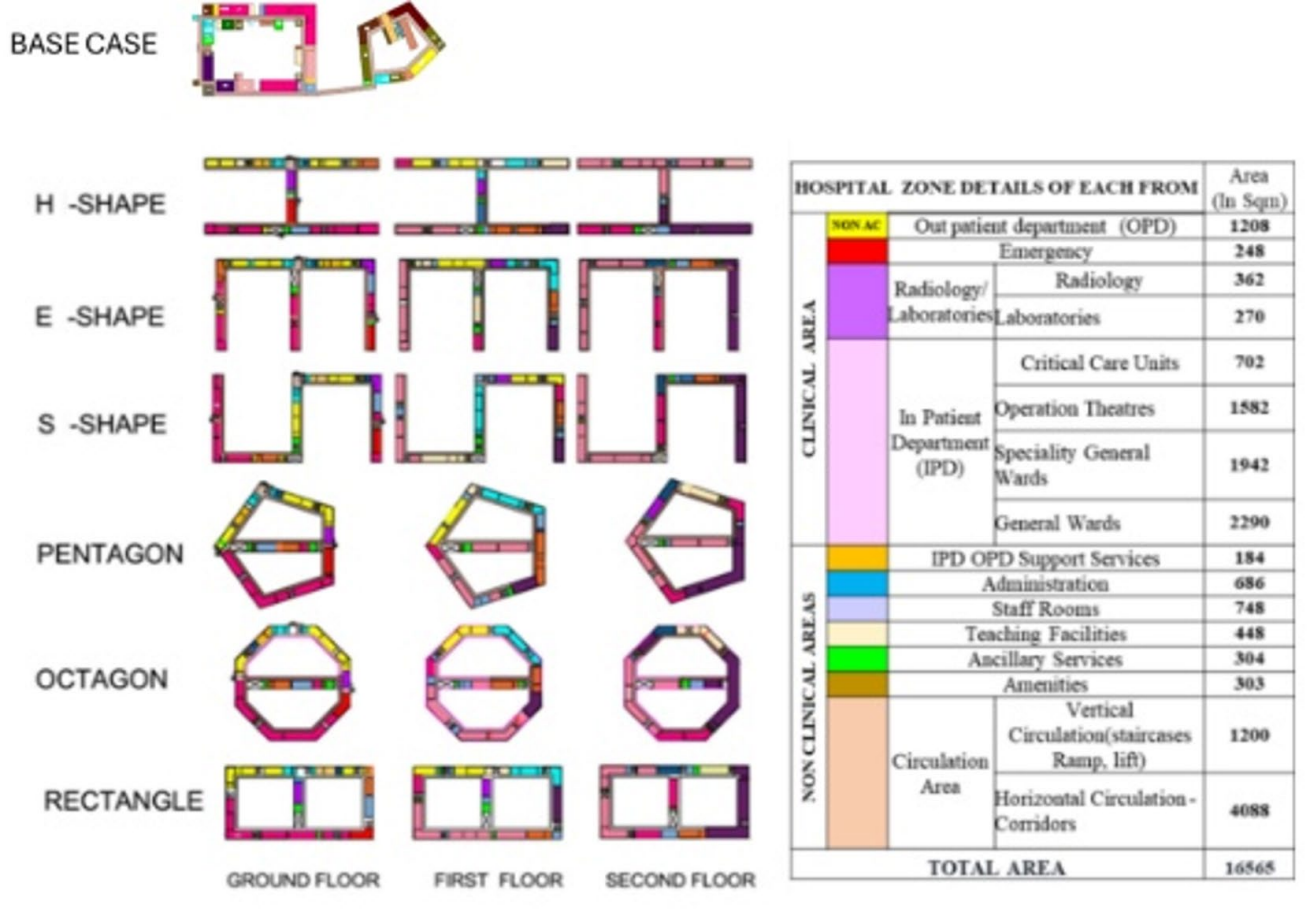
The 6 types of hospital forms with detailed space layout and area of different hospital zones.

This study adapted simulation-based strategy and the obtained data analysed through using statistical technique ANOVA (ANalysis of Variance). In this research there are 6 building forms with detailed space layout are developed to generate the simulation model through Design Builder software. Utilizing existing hospital design parameters and architectural principles, various layout configurations were generated to assess energy performance through simulations using advanced modelling software. Key metrics evaluated include cooling load, lighting demand, and overall energy consumption. Layout variants are influenced by factors such as reference buildings, design guidelines, climatic conditions, and functional needs, with each zone treated as a separate thermal zone. The study aims to analyze energy performance variations and develop optimized energy conservation strategies. The design includes low-rise structures with G + 2 floors, segregated main entrances, vertical cores comprising staircases, lift cores, and ramp cores. Modular grids are established for medical zones, with specific dimensions for wards and corridors, alongside detailed window and door specifications for different hospital areas.Building Energy Simulation is a useful tool for assessing a building’s performance based on its design and climate conditions^12^. The hospital forms and space layout variants are generated through the calibrated and validated reference district hospital building data located in the Mangalore, Karnataka, India a warm humid climate zone of India located in coastal region which is located 102 m above the sea level. The outside temperature ranges from 20.8 ◦C to 32.6 ◦C, with an average of 25.9 ◦C. Relative humidity ranges from 33.8% to 99.5%, with an average of 83%. Mangalore’s wind rose indicates that 16.78% of all wind directions originate in the west and south-west. February through May is considered the hot season, with daily highs typically reaching above 90◦F. In Mangalore, the duration of a day does not vary significantly from year to year; it remains constant at 53 min for every 12 h. In Mangalore, July is the cloudiest month of the year, with 90% of the sky being clouded or overcast on average. In Mangalore, the prevailing hourly average wind direction changes with the seasons. The All the input data derived based on the reference hospital function and working schedules. The forms are developed based on the warm humid climatic design with courtyards. Based on case studies of six government hospital layouts having more than 500 bed under the study frame from warm humid region of Karnataka state, India, the basic forms are created with various courtyard types such as semi closed, semi open and closed that are more climate-responsive and functional for the hospital in terms of space efficiency and climate responsiveness because the warm humid climate necessitates the importance of cross ventilation as a passive strategy. As part of this study there are 30 form variants are generated based on the openness of the courtyard. In these 18- closed courtyard forms, 8- semi closed courtyard forms and 4 semi open courtyard forms with single loaded corridors are developed. Through the preliminary analysis for basic hospital design guidelines and the building coverage only 14 forms are selected for the further daylight analysis using simulation software. From the daylighting analysis 5 forms are selected, which are H-shape, E-shape, S-shape, Octagon, Pentagon. All the areas of the forms are same as reference hospital building with 16565.0 sqm. Since the rectangular form with courtyard found to more adapted configurations in the hospitals of warm humid climate zone of India especially in coastal region, that shape also added in further development space layout of hospital. Finally, the 6-space layout/ form variants, H-shape, E-shape, S-shape, Octagon, Pentagon and Rectangle are considered as to develop base plans to obtain the optimal hospital building form with G + 2 floors in terms of energy consumption in Warm humid climate of India. The detailed methodology for this research shown in the Fig. 1. The space layout design for each form derived based on the functional requirement of the refence hospital as well as hospital design guidelines (Fig. 2). Hospital design guidelines as per National building code(2016) and requirements are crucial for creating aesthetically pleasing, functional, and efficient spaces that significantly impact patient outcomes, staff productivity, and satisfaction. General hospitals are classified into two major zones: clinical (outpatient department, inpatient departments, radiology and laboratories), and non-clinical (administration, teaching, and other amenities). The reference building is a low-rise structure with G + 2 floors and features single-loaded corridors, some of which are closed. There are three main entrances: a main entry, an emergency entry, and a service/IPD entry. The building includes six staircases, three lift cores, and two ramp cores. The design employs a repetitive grid system, with each module measuring 3 m x 8.5 m, structured to house 2 rows of beds within the general ward. Corridors are 2.75–3 m wide, with closed sections fitted with aluminum sliding windows, while other areas have parapet walls. The window designs vary: aluminum glazed sliding windows in air-conditioned areas with 50% opening, steel operable glazed windows in OPD areas with 90% opening, and wooden operable glazed windows in IPD areas also with 90% opening, all featuring external horizontal shading devices. Door specifications include aluminum doors with partial glazing in air-conditioned areas, wooden doors with glass panels in offices, and fully paneled wooden doors in IPD areas, alongside partially glazed wooden doors in OPD areas. Wall thicknesses are established at 600 mm for external walls, 300 mm for internal walls, and 230 mm for corridor walls, including parapets.

**Table 1:** The area statement of hospital Building form variants.

Layout variants	Base case	H-shape	E-shape	S-shape	Octagon	Pentagon	Rectangle
Area/floor (In sqm)	5522	5521	5520	5523	5523	5521	5523
Total area (G + 2 floors -In sqm)	16,565	16,564	16,561	16,569	16,568	16,563	16,569
Courtyard area	60%	70%	73%	73%	53%	55%	55%

Building areas are statistically analysed to find geometric efficiency and normalize size discrepancies, ensuring a fair baseline. In this study, the variability of hospital layout areas was measured using the sample standard deviation. The formula used considers the squared difference of each observation from the mean, aggregated and normalized by the sample size (*n* − 1) (Eq. 1). Taking the square root of this variance yields the standard deviation (Eq. 2).

1 $${\mathrm{Standard}}\:{\mathrm{variance}} - s = \sqrt {\frac{{\sum {\left( {x_{i} - \bar{x}} \right)^{2} } }}{{n - 1}}}$$

2 $${\text{Standard deviation }} - {\text{SD = }}\sqrt s$$

Further, to evaluate the extent of variation relative to the average total area, the coefficient of variation (CV) was calculated as the ratio of the standard deviation to the mean, expressed in percentage (Eq. 3).

3 $$CV = \frac{s}{{\bar{x}}} \times 100$$

The statistical results for the areas across the different layout variants (Table 1) through are as below,

Mean Total Area: 16,565.71 sqm.

- Variance (s): 10.97 sqm².
- Standard Deviation (SD): 3.31 sqm.
- Coefficient of Variation (CV): 0.02%.

Since the coefficient of variation (CV) is extremely small (0.02%) and negligible, there is no significant difference in the total areas across the various layout variants. This enables precise and significant comparisons in simulation outputs such as daylighting or energy use.

The design guidelines suggest optimal hospital design based on spatial closeness, connectivity, and adjacency requirements. It recommends emergency areas on ground floor, outpatient departments near main entrance, administration adjacent but separated, diagnostics centrally located, and pharmacy near outpatient and emergency departments. Wards should be close to other departments, with intensive care units near operating theatres. The study emphasizes balancing comfort and controllability in hospital design. Space layout variations in hospitals are shaped by existing reference buildings, design guidelines, climatic factors, and functional requirements. Effective energy modeling of these large multipurpose facilities requires consideration of their complex system composition and operational strategies for precise performance evaluation^30^. There are 77 functional zones, 6–8 toilet zones as well as corridors areas in each space layout variants of the hospital. Each zone is unique due to its various uses, occupancy, equipment and services detail. Broader zones are generated by grouping similar activities based on users, schedules, privacy levels, and functional requirements. The occupancy densities, plug loads, lighting densities for each zone are calculated as an input data for the Design Builder software. Reference buildings consist of low-rise structures with single-loaded corridors, segregated main entrances, vertical cores, and a building grid. Corridors are equipped with aluminium sliding windows in inpatient ward and office block, while windows in air-conditioned/ semi-air-conditioned areas are aluminium glazed sliding windows with 50% opening, steel operable glazed windows with 90% opening, and wooden operable glazed windows with 90% opening. The plug loads, occupancy densities along with construction materials are kept same for all the variants. The simulation process done for a calendar year 2023 and 8 different orientations.

The space layout variables like window to wall ratio and building height kept constant. The simulation also ran for monthly basis to get the EPI in kWh/m^2^/year.

EPI=Annual Energy Consumption (kWh)/Total Built-up Area (m^2^).

Where, Annual Energy Consumption (kWh)-Total electricity and fuel energy used by the facility in one year, converted to kilowatt-hours. Total Built-up Area (m²)- The gross floor area of the building measured in square meters. The EPI is expressed in kWh/m²/year.

Although several studies have focused on the relationship between building elements and EP, most of them analysed building and system design rather than building geometry^32^. The author utilized DesignBuilder software to simulate EP of space layout variants which is widely used due to its accuracy and user-friendly interface. DesignBuilder is a comprehensive user interface for EnergyPlus dynamic thermal simulation engine, organized into categories like model importing CAD, template components, material database. It provides advanced modelling tools for designing comfortable and energy-efficient building designs from concept to completion. DesignBuilder is a commercially available software package with a three-dimensional interface^33^. The evaluation of building EP through design builder software (Version 7.0.2.4) is crucial for optimizing building EP, aiming to find the best solution among various alternatives of the space layout in relation to building form and orientation^34^. The equipment schedules, lighting, air-conditioning schedules for each variants are followed as per the reference case hospital which the author collected during the walkthrough audit in the process of data collection as shown in the Tables 2 and 3. The weather data considered for the simulation is IND_ Karnataka_ Mangalore_ ISHRAE which is under ASHRAE climatic zone 1 A. The naturally ventilated functional zones are equipped with operable windows whereas the airconditioned zones are kept closed with sliding windows. The analysis highlights the significant role of building form in enhancing EP, specifically focusing on its impact on annual energy consumption and the EPI, which measures energy consumption per unit floor area. The 8 basic orientations are considered as a variable along with building forms. All the other features including infiltration rate, setpoint temperatures (As per the functional requirement), air flow rate kept constant.

**Table 2:** Activity, equipment and lighting schedules.

Hospital Zone Segregation		Timings	Working Hours	Occupancy	Lighting (L) Schedule	Computer (Comp) Schedule (Comp+Printer)	Office Equipment (OE) Schedule (AC + UPS)	Misc Equipment (Misc) Schedule
Outpatient Department (OPD)		9.00AM-5.00PM / 9.00AM-1.00PM	8	OPD-OCC-8 h	OPD-L-6 h	OPD -COMP-6 h	OPD -OE-6 h	OPD -MISC-2 h
		8.00–8.00		OPD-OCC-12 h	OPD-L-4 h			OPD -MISC-4 h
					OPD-L-2 h			
Emergency &Casualty ward(ECW)		24	24	IPD-OCC-24 h	ECW-L-14 h	ECW-COMP-10 h	ECW- OE- 8 h	ECW-MISC − 20 h
Radiology/ Laboratories	Radiology (RAD)	9.00AM-5.00PM / 9.00AM-1.00PM	8	RAD-OCC-8 h	RAD-L-2 h	RAD-COMP-6 h	RAD -OE -6 h	RAD -MISC − 1 h
	Laboratories (LAB)	9.00AM-5.00PM / 9.00AM-1.00PM	8	LAB-OCC-8 h	LAB-L -4 h	LAB-COMP-6 h	LAB-OE-8 h	LAB-MISC-24 h
						LAB-COMP-24 h		
In Patient Department (IPD)	Critical Care Units (CCU)	24		IPD-OCC-24 h	CCU-L-10 h	CCU-COMP-20 h	CCU-OE-20 h	CCU-MISC-20 h
		24			CCU-L-8 h		CCU-OE-6 h	CCU-MISC-6 h
								CCU-MISC-3 h
	Operation Theatres (OT)	7.30AM -7.30PM	12	OT-OCC-12 h	OT-L-6 h	OT-COMP-6 h	OT-OE-12 h	OT-MISC-24 h
							OT-OE-6 h	OT-MISC-3 h
							OT2-OE-12 h	OT-MISC-6 h
	Speciality General Wards	24	24	IPD-OCC-24 h	SGW-L-6 h		GW-OE-24 h	GW-MISC-20 h
							GW-OE-8 h	
							GW-OE-2 h	
	General Wards	24	24	IPD-OCC-24 h	GW-L-6 h	GW-COMP-24 h	GW-OE-24 h	GW-MISC-20 h
							GW-OE-8 h	
IPD /OPD Support Services (SS)		9.00–5.00	8	SS-OCC-8 h	SS -L-10 h	SS-COMP- 6 h	SS-OE-24 h	SS-MISC-18 h
		24	24	SS-OCC-24 h	SS -L-2 h	SS-COMP-24 h	SS-OE-8 h	SS-MISC-6 h
Administration Office (OFF)		9.00–5.00	8	OFF-OCC-8 h	OFF-L-6 h	OFF-COMP-6 h	OFF-OE-8 h	OFF-MISC-6 h
		7.30–7.30	12	OFF-OCC-12 h	OFF-L-2 h	OFF-COMP-12 h		OFF-MISC-8 h
					OFF-L-4 h			
Staff Rooms (ST)		24	24	ST-OCC-24 h	STF-L-14 h	ST-COMP-6 h	ST-OE-24 h	ST-MISC-2OHRS
		12	12	ST-OCC-12 h	STF-L-6 h	ST-COMP-24 h	ST-OE-8 h	ST-MISC-12 h
							ST-OE-6 h	
Teaching Facilities (TH)		9.00–5.00	8	TH-OCC-8 h	TEACH-L-6 h	TH-COMP-6 h-WD	TH-OE-2 h-WD	TH-MISC-6 h
					TEACH-L-4 h			TH-MISC-4 h
					TEACH-L-2 h			
Ancillary Services (ANS)		24	24	AS-OCC-24 h	ANS-L-6 h DN	ANS-COMP-6 h	ANS-OE-8 h	ANS-MISC-4 h
		7.30–7.30	12	AS-OCC-12 h	ANS-L-6 h		ANS-OE-6 h	ANS-MISC-12 h
								ANS-MISC-8 h
Amenities (AM)		24	24	AM-OCC-24 h	AM-L-4 h			CT-MISC-4 h
Circulation Area (COR)	Vertical Circulation	24	24	COR-OCC-24 h	COR-L- 12 h			COR-MISC-16 h
		8.00–8.00	12	COR-OCC-12 h	COR-L- 12 h			
	Corridors	24	24	COR-OCC-24 h	LIFT-L- 14 h			LIFT-MISC-14 h

HRS= HOURS.

**Table 3:** Calculated occupancy density, plug loads, lighting power densities for each variant.

Zones -Base Case		Occupancy Density			Lighting Density	Computers	Office Equipments
		Working Schedule		Occupancy Density	LPD (W/M2)	Power Density (W/M2)	Power Density (W/M2)
General Medicine/ Ortho OPD		8		0.14	4.73		44.05
Skin OPD		8		0.12	4.52	11.25	45.70
Urology OPD		8		0.23	11.94		295.71
Psychiatric OPD		8		0.18	6.36	10.91	20.45
Surgery OPD		8		0.12	12.12	55.21	54.47
Cardiac/ Neuro OPD		8		0.16	11.80		986.06
ECG				0.17	21.33	5.33	367.33
Ent OPD		8		0.10	5.18	88.38	285
Dental OPD		8		0.11	2.94	8.81	115.59
Eye OPD		8		0.16	6.87	7.95	203.74
Dressing /Sick Room		12		0.11	22.11		11.84
Emergency /Casualty Ward		24		0.09	33.35	6.78	39.48
Surgery Lab		8		0.38	15.24		108.57
Art & ICTC Lab				0.07	3.51	23.90	58.53
RNTCP Lab/Malaria Lab		8		0.59	14.12	381.18	548.82
Cd4 Lab				0.35	8.94	2.12	35.29
Radiology (X-Ray Scanning)-1		8		0.20	8.82	17.01	276.14
Radiology (X-Ray Scanning)-2		8		0.31	5.36		167.62
Radiology (OPD X-Ray)-3				0.17	5.11		677.91
ICU		24		0.10	10.62	11.54	923.07
Cardiac ICU		24		0.11	27.17	90.57	566.037
Post-Operative Ward		24		0.15	17.80		40
Nurse Station/Toilet		24		0.12	10.21		12.72
Post-Operative Ward –Female/Male							
Dialysis Ward		24		0.08	20.28		827.58
Dialysis Rest Room		24		0.25	1.46		
Medication Room/OT Storeroom		12		0.06	7.86		50
Endoscopy Room		12		0.22	16.11	26.67	666.66
OT Complex − 1		12		0.03	14.33	12.24	485.57
OT Complex − 2		24		0.04	14.27	9.19	228.37
Jail Ward_Male & Female		24		0.08	29.39		
Palliative Ward		24		0.14	13.91		
General Ward Ward − 1 _Medical /Cardiac		24		0.21	14.55		
General Ward − 2_Psychiatric-Female		24		0.12	16.71		
General Ward − 3a _Psychiatric-Male		24		0.15	18.69		36.36
General ward- 4 _Ortho-Female		24		0.14	25.20		
General Ward- 5 _Ortho-Male		24		0.16	11.11		10.00
General Ward _Eye -Female		24		0.21	9.67		109.59
General Ward 17 _Surgery -Female		24		0.14	10.90		19.70
Male Surgery Ward − 2		24		0.14	10.20		9.11
General Ward- 6		24		0.18	26.42		
General Ward-7		24		0.15	14.65		
General Ward-8		24		0.17	6.87		9.39
General Ward-9		24		0.16	11.32		
General Ward-10 + 11		24		0.14	15.25	8.65	36.04
General Ward-12 + 13		24		0.14	13.40	9.46	
General Ward-14		24		0.16	14.26		37.04
General Ward-19		24		0.17	6.81		
General Ward 21		24		0.11	6.21		
General Ward 22		24		0.14	14.18		
General Ward 23		24		0.18	13.82		
General Medicine/ Ortho OPD	8		0.14		4.73		44.05
Skin OPD	8		0.12		4.52	11.25	45.70
Urology OPD	8		0.23		11.94		295.71
Psychiatric OPD	8		0.18		6.36	10.91	20.45
Surgery OPD	8		0.12		12.12	55.21	54.47
Cardiac/ Neuro OPD	8		0.16		11.80		986.06
ECG			0.17		21.33	5.33	367.33
Ent OPD	8		0.10		5.18	88.38	285
Dental OPD	8		0.11		2.94	8.81	115.59
Eye OPD	8		0.16		6.87	7.95	203.74
Dressing /Sick Room	12		0.11		22.11		11.84
Emergency /Casualty Ward	24		0.09		33.35	6.78	39.48
Surgery Lab	8		0.38		15.24		108.57
Art & ICTC Lab			0.07		3.51	23.90	58.53
RNTCP Lab/Malaria Lab	8		0.59		14.12	381.18	548.82
Cd4 Lab			0.35		8.94	2.12	35.29
Radiology (X-Ray Scanning)-1	8		0.20		8.82	17.01	276.14
Radiology (X-Ray Scanning)-2	8		0.31		5.36		167.62
Radiology (OPD X-Ray)-3			0.17		5.11		677.91
ICU	24		0.10		10.62	11.54	923.07
Cardiac ICU	24		0.11		27.17	90.57	566.037
Post-Operative Ward	24		0.15		17.80		40
Nurse Station/Toilet	24		0.12		10.21		12.72
Post-Operative Ward –Female/Male							
Dialysis Ward	24		0.08		20.28		827.58
Dialysis Rest Room	24		0.25		1.46		
Medication Room/OT Storeroom	12		0.06		7.86		50
Endoscopy Room	12		0.22		16.11	26.67	666.66
OT Complex − 1	12		0.03		14.33	12.24	485.57
OT Complex − 2	24		0.04		14.27	9.19	228.37
Jail Ward_Male & Female	24		0.08		29.39		
Palliative Ward	24		0.14		13.91		
General Ward Ward − 1 _Medical /Cardiac	24		0.21		14.55		
General Ward − 2_Psychiatric-Female	24		0.12		16.71		
General Ward − 3a _Psychiatric-Male	24		0.15		18.69		36.36
General ward- 4 _Ortho-Female	24		0.14		25.20		
General Ward- 5 _Ortho-Male	24		0.16		11.11		10.00
General Ward _Eye -Female	24		0.21		9.67		109.59
General Ward 17 _Surgery -Female	24		0.14		10.90		19.70
Male Surgery Ward − 2	24		0.14		10.20		9.11
General Ward- 6	24		0.18		26.42		
General Ward-7	24		0.15		14.65		
General Ward-8	24		0.17		6.87		9.39
General Ward-9	24		0.16		11.32		
General Ward-10 + 11	24		0.14		15.25	8.65	36.04
General Ward-12 + 13	24		0.14		13.40	9.46	
General Ward-14	24		0.16		14.26		37.04
General Ward-19	24		0.17		6.81		
General Ward 21	24		0.11		6.21		
General Ward 22	24		0.14		14.18		
General Ward 23	24		0.18		13.82		

### Results and analysis

**Fig. 3:**
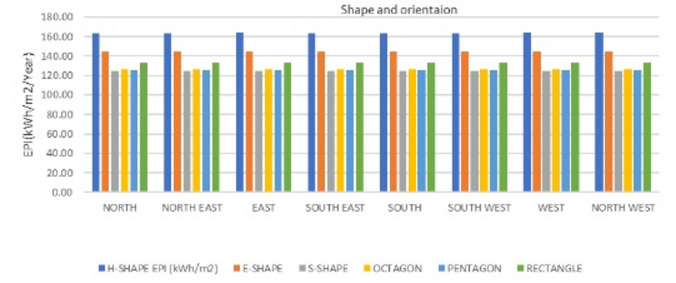
EPI of 6 space layout variants in relation to orientation.

The different building forms are simulated through DesignBuilder software keeping the all plug loads, construction details and occupancy details constant. The EPI of the developed hospital building forms as shown in the figure two are simulated with constant plug loads, construction details, activity details are obtained as shown in the Fig. 3. Month-wise analysis provides critical insights into the temporal variations in energy needs, enabling adaptive design solutions that cater to the unique demands of each season. Such studies are particularly relevant where high humidity and rainfall during the monsoon necessitate specific strategies for moisture control and ventilation efficiency.

**Fig. 4:**
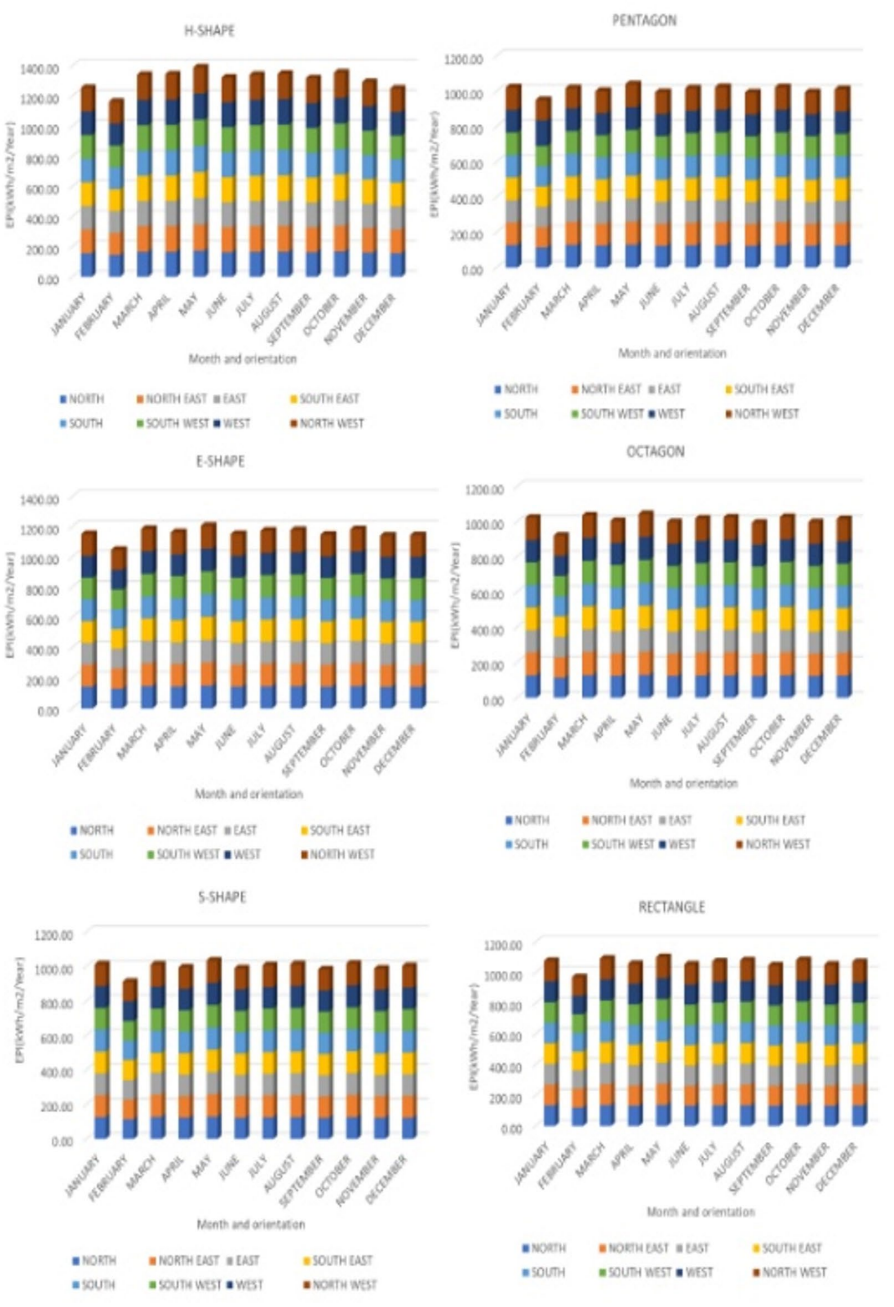
Monthly EPI of 6 space layout variants in relation to orientation.

Additionally, the findings can inform sustainable design practices that reduce energy dependence, particularly during periods of extreme heat or high humidity. Thus, the development of climate-responsive architecture that balances energy efficiency with occupant comfort of the hospital, tailored to the dynamic climatic conditions of warm humid climate of India necessitates this study. The monthly variation of EP in relation to form and orientation based on the simulation data for one year in the hospital space layout variants is shown in Fig. 4.

The two-way ANOVA results reveal that the orientation and form of buildings significantly influence the EPI across all seasons in India’s warm humid climatic region, although the magnitude of their effects varies.

Forms consistently exhibits a highly significant main effect (Probability value (p) < 0.001) throughout the year, indicating its dominant role in determining EPI (Table 4). In contrast, the significance of orientation varies, with notable impacts in certain months, such as the post-monsoon season (e.g., October: F (Ratio of the mean square for the between groups divided by the mean square within groups) = 2.34, *p* = 0.045; November: F = 4.82, *p* = 0.001), while being less impactful in other months, such as December (F = 0.90, *p* = 0.52).During the winter season (January - February), the combined influence of orientation and form explains nearly all the variation in EPI (R² = 99.99%), with form contributing substantially more than orientation (F = 104376.03 vs. F = 4.36).

**Table 4:** Influence of orientation and form of the Building on EPI.

Month	Standard error *R*-Sq =*R*^2^ *R*-Sq(adj)= *R*^2^ Adjusted
January	0.105 99.99% 99.99%
February	4.322 89.93% 86.48%
March	2.944 97.18% 96.21%
April	0.293 99.97% 99.97%
May	0.186 99.99% 99.99%
June	0.302 99.97% 99.96%
July	0.085 98.77% 96.37%
August	0.092 96.45% 92.59%
September	0.085 98.37% 94.69%
October	0.069 94.76% 92.51%
November	0.077 94.32% 90.62%
December	0.077 96.27% 93.68%

Similarly, during the summer months (March - May), building form remains the predominant factor (F = 27596.27 in April and F = 71135.73 in May), while orientation’s contribution is relatively minor and statistically insignificant (*p* = 0.49 and *p* = 0.344). In the monsoon season (June - September), form continues to dominate the variance in EPI, with extremely high F-values (F > 23438) and *p* < 0.001. Orientation, however, exhibits weaker effects, with non-significant results in June (F = 0.86, *p* = 0.548) and August (F = 0.70, *p* = 0.67). This can be attributed to the high levels of rainfall and humidity reducing the relative influence of building orientation on energy efficiency during this period. Interestingly, in the post-monsoon season (October - December), orientation becomes more critical, with significant results in October (F = 2.34, *p* = 0.045) and November (F = 4.82, *p* = 0.001). This shift may be due to changes in solar exposure and wind patterns as the region transitions to drier and more stable climatic conditions. The model consistently achieves high R² (Percentage of the total variance of all the data, combining all the groups, that may be attributed to variations in the group means) values across all seasons, indicating that the combination of building form and orientation effectively explains the variation in EPI. The highest R² values occur during the summer months (R² = 99.97% in April and R² = 99.99% in May), where form’s influence is exceptionally pronounced. Conversely, lower R² values during the winter months (R² = 89.93% in February) and post-monsoon season (R² = 94.76% in October) suggest greater unexplained variation, potentially due to additional environmental or operational factors not included in the model. These findings underscore the importance of considering courtyard building form as the primary determinant of EPI, with orientation playing a supplementary role, particularly in post-monsoon months where it becomes more significant. This highlights the necessity of integrating both courtyard form and orientation in energy-efficient building designs tailored to the unique warm humid climatic conditions of Coastal India.

During the post-monsoon season, optimal building orientation in hospitals is crucial for enhancing solar heat gain and ventilation due to drier and more stable climatic conditions. The findings underscore the importance of considering orientation in energy-efficient hospital designs during transitional periods like October and November^35^. The above findings have necessitated the study of the interaction effect of form and orientation of the hospital building with courtyards on the EPI which is discussed in the next section.

## The influence of different Building forms and orientations of the Building on EP

The influence of form and orientation of the building on the EPI due to seasonal variation throughout the year studied in the previous section has necessitated the testing of the hypothesis in relation to these associations to provide empirical evidence for relationships between these variables.

**Fig. 5:**
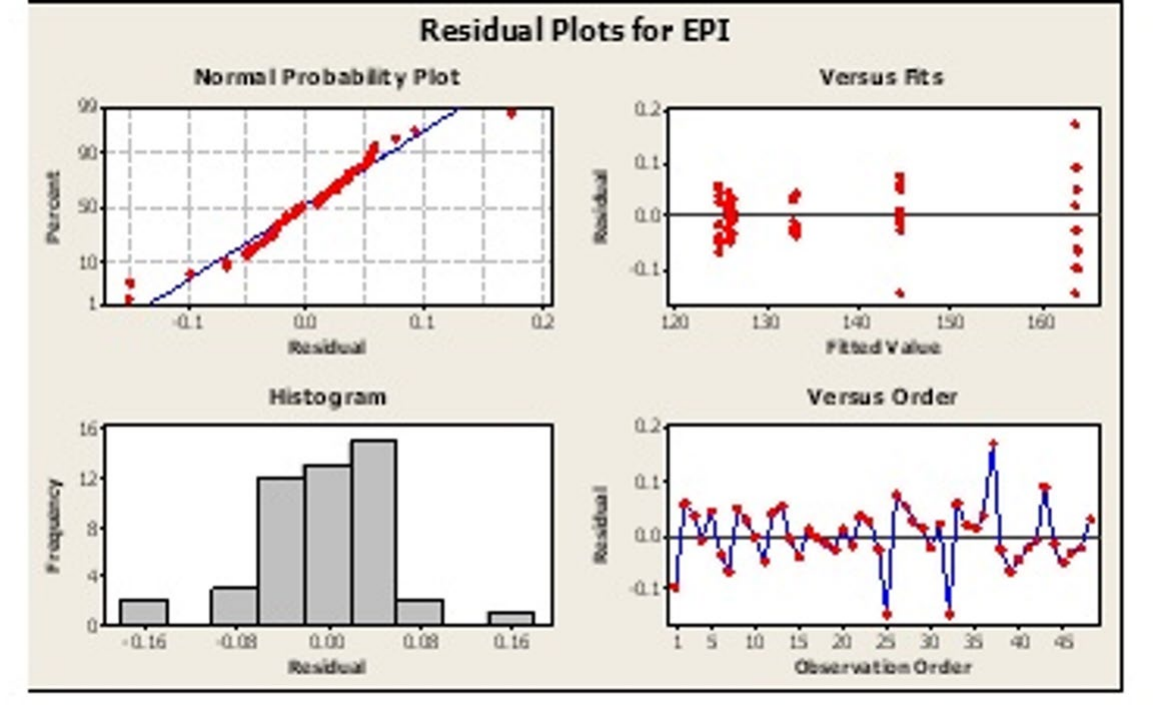
Residual plots of EPI.

Further, the interaction of building forms and orientation significantly affects energy performance (EP) by influencing heat gain, natural lighting, and ventilation efficiency. Certain combinations, like elongated forms aligned with prevailing winds, improve natural ventilation and decrease cooling energy needs^36^. Furthermore, optimal orientations for compact forms can minimize solar exposure on larger facades, demonstrating that synergistic design considerations improve overall energy efficiency^37^. However, there is no adequate empirical proof to support the relationships between these variables and their combined effect on EPI and hence the following hypotheses have been postulated.

H1: There is a significant effect of the form of the building on EPI.

H2: There is a significant effect of the orientation of the building on EP I.

H3: There is a significant effect of the interaction of form and orientation of the building on EPI.

Residual Plots of EPI: The software used for Two-way ANOVA and Regression analysis is Minitab and the output are shown in Fig. 5. The residual plots provide the descriptive of the EPI data. First of all, normality of the data is an important requirement of the ANOVA as it affects the validity of the results. In the present case, it can be observed that except for the three outliers the data closely follows the normal line indicating that the residuals are by and large normal.

**Fig. 6:**
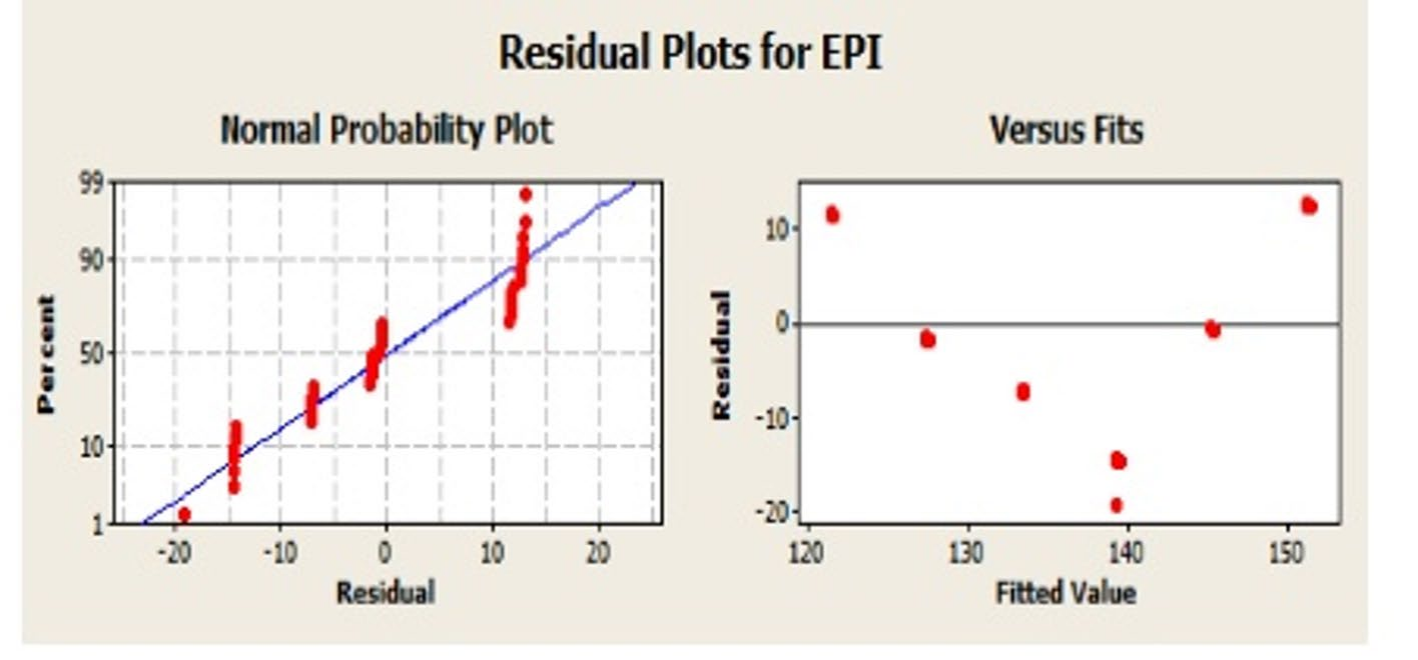
Residual plots of EPI showing the interaction effect of forms and orientation.

Residuals versus Fitted Values Plot with the random scatter further justifies the normality of the data. A roughly bell-shaped histogram spread closely to zero between − 0.16 to + 0.16 (Fig. 6) with slight skew towards the right can also be observed that supports the ANOVA assumptions further.

Finally, the Residuals vs. Observed Order Plot shows a random scatter without trends indicates no autocorrelation. Thus, the data supports the assumptions of the ANOVA and the data may be subjected to inferential statistics.

## Variance of EPI based on forms and orientation

The two-way ANOVA results indicate that both building forms and orientation significantly influence the EPI, thus supporting the hypotheses H2 and H3. The main effect of forms is highly significant (F = 161.86, *p* < 0.001) (Table 5), with a substantial sum of squares (SS = 2719.2), accounting for the majority of the variation in EPI. Orientation also has a statistically significant effect (F = 8.68, *p* < 0.001;), though its contribution (SS = 204.12) is smaller compared to forms. The error term reflects minimal unexplained variation (SS = 117.6), indicating a well-fitted model. The total variation in EPI (SS = 3040.92) is predominantly explained by the two factors, as evidenced by the high R-Squared value (R^2^ = 96.13%) and adjusted R-Squared (R^2^adj = 94.81%). The low standard error (S = 1.833) further supports the reliability of the model. These findings underscore the critical roles of both forms and orientation in optimizing building EP, with forms emerging as the dominant factor.

**Table 5:** Two-way ANOVA of EPI based on forms and orientation of Buildings.

Source	DF	SS	MS	F	*P*
Forms	5	2719.2	543.84	161.86	0.001
Orientation	7	204.12	29.16	8.68	0.001
Error	35	117.6	3.36		
Total	47	3040.92			
SE = 1.833 R-Sq = 96.13% R-Sq (adj) = 94.81%					

## Interaction effect of forms and orientation on the EPI

The regression model demonstrates the significant influence of the interaction between form and orientation of the building (H3), in addition to the significant influence of form and orientation individually. The constant term (158.94) represents the baseline EPI when both predictors are at zero. forms have a strong negative main effect on EPI (*β* = −6.171, *p* < 0.00) (Table 6), indicating that as the building form variable increases, EPI decreases significantly. Orientation also has a statistically significant negative main effect (*β* =−0.5, *p* = 0.00), even though smaller, showing that EPI reduces slightly with increasing orientation values. Importantly, the interaction between forms and orientation (*β* = 0.4, *p* < 0.00) is highly significant, suggesting that the combined effect of forms and orientation on EPI is not additive but interdependent.

The model explains 98.6% of the variation in EPI, with an adjusted R^2^ of 98.5%, indicating an excellent fit. The low standard error (S = 0.998) further supports the model’s accuracy in predicting EPI. These findings highlight that optimizing both forms and orientations, while considering their interaction, is crucial for achieving energy-efficient building designs. Thus, we can conclude that all the three hypotheses are supported. The t-statistic is a statistical measure used in regression analysis to determine the significance of a regression coefficient. It is calculated by dividing the coefficient by its standard error, and a large absolute value indicates a significant difference from zero. The t-statistic is often used alongside the p-value for hypothesis testing.

**Table 6:** Interaction effect of forms and orientation on the EPI.

Predictor	β (Constant)	SE Coeff	t	*p*
Constant	158.94	0.72	219.4	0.00
Forms	-6.171	0.19	-33.18	0.001
Orientation	-0.5	0.14	-3.49	0.00
Forms*Orientation	0.4	0.037	10.86	0.00

S = 0.998 R-Sq = 98.6% R-Sq(adj) = 98.5%.

The regression equation for the model is as follows.

Dependent variable = Constant + Form constant x Forms +orientation constant x Orientation+ Form *orientation constant x Forms*Orientation.

EPI = ***β*** + ***β1*** X Forms + ***β*2** X orientation + ***β3*** X Forms*Orientation.

EPI = 158.94–6.171 x Forms − 0.5 x Orientation + 0.4 x (Forms*Orientation).

## Discussions

The consistent significance of form in determining the EPI and the varying influence of orientation lies in the physical properties of buildings and the climatic patterns specific to Coastal region of India throughout the year. form governs the building’s surface area-to-volume ratio, which has a direct impact on heat exchange. Compact forms minimize heat gain or loss, while elongated building forms influence natural ventilation and airflow efficiency in a hospital building which are very essential as functional requirements especially in naturally ventilated government hospitals^31^. These properties are fundamental and remain significant throughout the year, explaining the consistently high statistical significance of form. Building form determines the effectiveness of passive design strategies, such as shading, thermal mass, and natural cooling^38^. These strategies are crucial in regulating EP across all climatic conditions, making hospital building form the dominant factor irrespective of seasonal variations. The impact of building form is resilient to changing seasons, as it directly influences internal thermal comfort and energy needs for cooling or heating, irrespective of external factors like solar radiation or wind direction^39^.

### Varying influence of orientation

During the post-monsoon season, high humidity levels necessitate effective ventilation and optimized building forms to ensure comfort and reduce energy consumption. These design principles enhance energy efficiency in hospital buildings, leading to increased sustainability and cost-effectiveness, while also improving occupant comfort and health outcomes^40^.

## Combined insights of Building form and orientation

Form is essential in environmental planning (EP), impacting key design aspects such as heat retention, natural cooling, and energy needs throughout the year. The orientation of buildings is also affected by external climatic elements, including solar intensity and wind direction^39^. Building orientation and form significantly influence energy efficiency and thermal performance in Coastal India, especially during post-monsoon seasons. While orientation’s effect varies with solar and wind patterns, building form becomes more critical for energy performance in winter and summer climates. For hospitals, a focus on form is essential to minimize heat loss and enhance passive solar heating, particularly due to intensified summer solar conditions. Compact forms are recommended to reduce solar heat gain, while larger forms lead to increased energy consumption. Overall, the study highlights the predominance of building form over orientation in warm, humid coastal regions, underscoring strategies like roof shading and insulation to manage heat gain^41^. Monsoon weather in Coastal India creates consistent cloud cover and low solar radiation, reducing the significance of hospital building orientation on energy performance index (EPI). During heavy rain and high humidity, the emphasis shifts to thermal comfort and air quality, making orientation strategies less relevant. Energy use is primarily concentrated on humidity control through dehumidification or natural ventilation^42^. Hospital building form greatly affects energy performance index (EPI) during monsoon months through rainwater management, humidity control, and ventilation. In post-monsoon Coastal India, orienting buildings to optimize solar heat gain and natural ventilation minimizes cooling loads by reducing afternoon sun exposure and aligning with prevailing winds, emphasizing the importance of building design in meeting energy demands^43,44^.

The hypothesis testing (H3) has revealed the interactions effect between building form and orientation in producing the combined effect on EPI. This finding is rather unique to this research as not many researchers have studied the interaction effects of variables on the EPI.

Following are the excerpts from the hypotheses testing:

- Form consistently influences hospital building EP across all seasons due to its impact on surface area-to-volume ratios, heat retention, and ventilation.
- Orientation’s role is context-dependent, becoming significant during certain seasons like post-monsoon when solar heat gain and wind-driven ventilation are critical.
- The interaction effect of building form and orientation highlights the importance of combining these factors for optimal energy efficiency.
- Seasonal analyses (winter, summer, monsoon, post-monsoon) emphasize tailored design strategies that account for climatic variations in coastal India.

These points encapsulate the practical implications urban designers can draw from the EP analysis.

## Optimum design

On considering the interaction effects of forms and orientation of buildings the following three combinations emerge out to be the most optimal designs. Among these three combinations, S-shape building form with West, North-West, or South-West orientation with EPI value of 124.8 kWh/m^2^/Year (Table 7) will be the most ideal combination considering the following aspects.

Among the three orientations, S-shape building form with North-west has been selected due to the following considerations though the EPI remains the same for all the three:

### Optimized solar heat gain

The North-West orientation effectively minimizes solar heat gain in hospital buildings, especially in coastal and tropical regions where West and South-West orientations face high afternoon sun exposure. This approach helps maintain thermal comfort in naturally ventilated wards by reducing excessive heat during peak afternoon hrs^44^. Also confirmed that North-West facing buildings experience lower solar exposure during the hottest part of the day, further contributing to reduced cooling needs^45^.

**Table 7:**
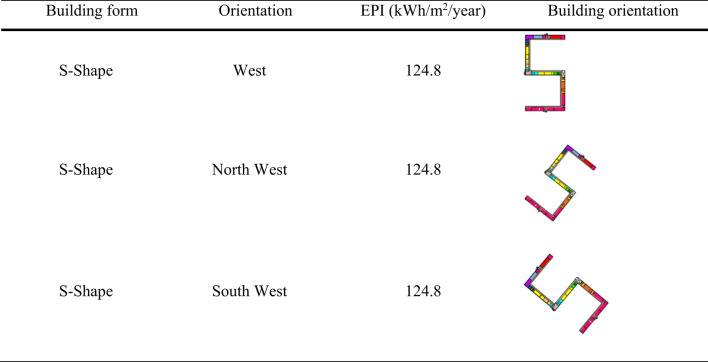
Optimal design options

### Enhanced natural ventilation

In coastal India, the prevailing wind patterns during post-monsoon and summer seasons align with North-West orientations, optimizing natural ventilation and reducing reliance on mechanical cooling in the hospital buildings. Aligning building orientation with prevailing winds enhances airflow, making the North-West orientation particularly efficient in capturing breezes^46^. The buildings oriented towards the North-West facilitate better cross-ventilation, leading to more effective cooling during transitional and summer seasons compared to West or South-West orientations^40^.

### Climate responsiveness

The North-West orientation effectively balances minimizing peak afternoon solar heat gain and enhancing ventilation in tropical climates, which experience high humidity and seasonal wind shifts. This orientation reduces solar exposure while improving airflow^47^. North west orientation optimizes both diffused light and wind-driven cooling, making it particularly advantageous in regions like coastal India, where both solar intensity and humidity levels fluctuate^48^.

### Comfort during transitional seasons

North-West orientations also provide enhanced thermal comfort during transitional seasons, such as post-monsoon. Fang et al., demonstrated that these buildings benefit from diffused solar radiation and consistent wind exposure, crucial for balancing natural lighting and ventilation^49^. North-West-facing buildings improve indoor comfort by aligning with seasonal wind patterns, offering a more pleasant indoor environment during the transitional periods^38^.

### Spatial design implication

The analysis shows that in warm, humid climates, buildings with façades facing southwest and west optimize airflow due to prevailing winds. Natural ventilation areas, such as wards and outpatient departments, are situated in the northwest to west to enhance thermal comfort and passive performance. Air-conditioned units like operation theatres and intensive care units are placed on the east and north sides to minimize solar gains and mechanical cooling. The study assesses large hospital designs, highlighting a zoning trend where naturally ventilated spaces are on the south, west, southwest, and northwest façades, with corridors providing shade and cooler passage on the inner courtyard side. Air-conditioned zones, such as operating theatres, ICUs, and diagnostic suites, are strategically positioned to minimize direct solar gain. In cases facing the west or south, buffer zones are incorporated to reduce heat infiltration. Naturally ventilated zones remain open or semi-open, responding directly to the outdoor climatic conditions of warm-humid regions. The study suggests that hospital planning should strategically integrate both passive and active environmental control measures. Courtyard planning supports microclimatic regulation by enhancing shading, encouraging natural airflow, and providing thermally comfortable communal spaces. Linear configurations allow for controlled orientation and zoning, ensuring each function benefits most from its environmental context. This integrated approach can serve as a guiding principle for future healthcare facility designs in similar climatic conditions.

### Practical implications

Based on the discussions and hypothesis testing, following are the practical implications for urban designers in coastal India which are also supported well with earlier research findings in many different countries with similar climatic conditions:

Prioritize compact building forms: Research findings indicate that compact hospital building forms are essential for reducing surface area-to-volume ratios, thereby minimizing heat gain and loss. This design approach enhances energy efficiency year-round by improving heat retention in winter and reducing cooling needs in summer. Evidence shows that these compact forms effectively reduce energy demands through minimized heat exchange with the external environment, leading to optimized energy and functional efficiency across seasons^50^.

Leverage elongated building forms: The findings indicate that in hot and humid climates, elongated building forms aligned with prevailing wind directions significantly enhance natural ventilation and airflow efficiency, which is critical for reducing cooling loads during the summer months^46^. This reduces the reliance on mechanical cooling in tropical climates.

Strategically optimize orientation: Research indicates that buildings should be oriented to reduce solar heat gain in summer, ideally with north-south facades, while optimizing solar exposure in winter for better heating. After the monsoon, building orientation should be aligned to facilitate consistent wind patterns and natural ventilation^43^. This dual approach enhances seasonal energy efficiency by optimizing orientation in humid climates, especially during transitional seasons like post-monsoon. It effectively reduces energy consumption through wind-driven ventilation and solar exposure management^51^.

Focus on seasonal variations: This dual approach enhances seasonal energy efficiency by optimizing orientation in humid climates, especially during transitional seasons like post-monsoon. It effectively reduces energy consumption through wind-driven ventilation and solar exposure management^52^. For summer, elongated building forms paired with shading devices play a crucial role in minimizing solar heat gain and improving ventilation^50^. In monsoon seasons, moisture management and natural cross-ventilation are key challenges. The strategically placed windows and moisture-resistant materials will manage internal moisture^39^. Optimizing building orientation during the post-monsoon transition is essential for maximizing solar heat gain and utilizing wind for natural ventilation, significantly reducing the need for mechanical cooling^49^.Aligning building design to maximize solar exposure and leverage cooling breezes fosters both comfort and energy efficiency^53^.

Utilize passive design elements: The research emphasizes the need for passive cooling strategies such as shading devices, reflective materials, and thermal insulation to minimize reliance on active cooling systems. These strategies should be adapted according to the building’s form, orientation, and the functional requirements of hospital zones^54^. Further, thermal insulation and reflective materials improve energy efficiency and reduce cooling demands, particularly in climates where passive cooling strategies are integrated with the building’s form and orientation^55^.

Account for interaction effects: The results highlight the significance of the combined influence of form and orientation on energy performance. Elongated forms aligned with prevailing wind directions enhance natural ventilation and energy efficiency, emphasizing the importance of synergistic design considerations in reducing cooling loads^46^. The building form and orientation interact to modulate heat gain and ventilation efficiency^37^. Elongated forms aligned with wind directions were found to significantly improve EP in warm humid climates.

Adapt designs to local climatic conditions: This research highlights the distinct climatic conditions of Coastal India, characterized by high humidity, significant rainfall, and seasonal wind variations. It underscores the need for climate-adaptive building designs, such as cross-ventilation via strategically placed windows and openings, to improve airflow, reduce indoor humidity, and enhance comfort^46^; Overhangs and shading devices that protect walls from direct rain impact and minimize solar heat gain, reducing cooling demands^56^; buildings oriented to align with prevailing wind directions and minimize solar exposure on large facades to optimize natural ventilation and reduce heat gain^47^. These features address key challenges in coastal climates, such as humidity, rainfall, and wind, ensuring sustainable and resilient building performance.

Promote energy-efficient facade design: The study suggests that facades should be designed to optimize natural lighting and reduce solar heat gain, especially during transitional seasons. This can be achieved through Dynamic Shading Systems, which control light penetration and shading based on solar intensity^55^; Horizontal or angled light shelves that direct natural light deeper into indoor spaces while shading the lower portions of windows from direct sunlight^49^; Green Facades with Vegetation reduces solar heat gain by shading and cooling the facade while allowing diffused natural light to penetrate^57^; Parametric Facade Systems with irregular perforations that balance natural light penetration and shading based on solar exposure and wind conditions^38^. The design of facades in Coastal India aims to maximize natural lighting while minimizing solar heat gain, promoting energy efficiency and climate responsiveness.

## Conclusions

The study emphasizes the importance of courtyard building form as the primary determinant of EP, with orientation playing a supplementary role, especially in post-monsoon months, in energy-efficient hospital designs for the warm humid climate of coastal India. The study reveals a significant interaction between forms and orientation, indicating an interdependent effect on energy-efficient building design. The model explains 98.6% of variation in EPI, with an adjusted R^2^ of 98.5%. The low standard error supports its accuracy in predicting EPI. Optimizing both forms and orientations is crucial for achieving energy-efficient building designs. The North-West orientation with an S- shape building form with semi closed courtyard offers superior energy efficiency, thermal comfort in hospital buildings in coastal region of India compared to West and South-West orientations and is strongly recommended based on the findings of this research. The naturally ventilated hospital zones especially general wards, outpatient departments can be exposed directly to the north west or west orientation to gain the benefit of natural ventilation and airconditioned zones like operation theatres, intensive care units can be placed in such a way that to reduce solar gains by integrating passive design elements, such as shading devices, reflective materials, and thermal insulation. Semi-closed courtyard building forms, such as S- or H-shapes, are effective in enhancing natural ventilation and improving environmental performance. The GRIHA rating system promotes compact design but does not dictate specific building typologies. By utilizing semi-enclosed courtyard designs with an ideal height-to-width ratio of approximately 2:1, architects can achieve an optimal balance between ventilation and daylighting. For architects and urban planners operating in warm-humid climates, these findings provide insightful information. EP can be greatly enhanced by dynamically including shape and orientation into the design process. For public infrastructure to be sustainable, this strategy encourages the construction of passive, climate-responsive hospitals and institutional buildings. Integrating sustainable building practices into planning and policy is crucial for addressing climate change. By focusing on passive design, adaptive orientation, and low-energy building forms, decision-makers can support climate action, lower carbon emissions, and promote the development of resilient, net-zero communities. Further research into innovative forms and their orientation strategies will enhance sustainable design practices especially in large government hospitals with major concentrations on naturally ventilated spaces like general wards, outpatient areas as well as public spaces. One limitation of this work is the analysis done only based on the building EP index in relation to building form and orientations whereas further investigation is necessary to explore the effects of various space layout variables like WWR, surface area, positioning of hospital functional zone, shading parameters to understand the comprehensive space layout influence on energy, solar gain as well as daylighting performance in hospital buildings of warm humid climate of Coastal India.

## Data Availability

The data that support the findings of this study are openly available in Figshare at [https://doi.org/10.6084/m9.figshare.30095311.v1](https:/doi.org/10.6084/m9.figshare.30095311.v1) , Reference number -251461190 #TrackingId:23575385.
